# Antibacterial and antibiofilm activity of halogenated phenylboronic acids against *Vibrio parahaemolyticus* and *Vibrio harveyi*

**DOI:** 10.3389/fcimb.2024.1340910

**Published:** 2024-03-28

**Authors:** Ezhaveni Sathiyamoorthi, Jin-Hyung Lee, Jintae Lee

**Affiliations:** School of Chemical Engineering, Yeungnam University, Gyeongsan, Republic of Korea

**Keywords:** antibacterial, antibiofilm, halogenated acids, *Vibrio harveyi*, *V. parahaemolitycus*

## Abstract

*Vibrios* are associated with live seafood because they are part of the indigenous marine microflora. In Asia, foodborne infections caused by *Vibrio* spp. are common. In recent years, *V. parahaemolyticus* has become the leading cause of all reported food poisoning outbreaks. Therefore, the halogenated acid and its 33 derivatives were investigated for their antibacterial efficacy against *V. parahaemolyticus*. The compounds 3,5-diiodo-2-methoxyphenylboronic acid (DIMPBA) and 2-fluoro-5-iodophenylboronic acid (FIPBA) exhibited antibacterial and antibiofilm activity. DIMPBA and FIPBA had minimum inhibitory concentrations of 100 μg/mL for the planktonic cell growth and prevented biofilm formation in a dose-dependent manner. Both iodo-boric acids could diminish the several virulence factors influencing the motility, agglutination of fimbria, hydrophobicity, and indole synthesis. Consequently, these two active halogenated acids hampered the proliferation of the planktonic and biofilm cells. Moreover, these compounds have the potential to effectively inhibit the presence of biofilm formation on the surface of both squid and shrimp models.

## 1 Introduction

Vibriosis is a prevalent disease in aquaculture that significantly impacts fish, crustaceans, and mollusk species (Baker-Austin et al., 2018). *Vibrio* species, including *Vibrio parahaemolyticus*, *Vibrio harveyi*, and *Vibrio vulnificus* (Lee et al., 2022), are distributed widely in marine environments worldwide and are recognized as the primary etiological agents of Vibriosis (Igbinosa and Okoh, 2008). The pathogenicity of *Vibrio* spp. has been associated primarily to the formation of biofilm and the occurrence of various virulence factors, such as lipopolysaccharides, polysaccharides, flagella, and cytotoxins. The cell signaling process known as quorum sensing which is responsible for controlling the activity of these components (Urmersbach et al., 2015). Microbial biofilm formation has become catastrophic risk in many food-processing surroundings due to high antimicrobial tolerance. The presence of high moisture content, availability of nutrients, and microbes existing in the raw materials contribute to the establishment of biofilms, which may cause to food putrefaction and can facilitate the spread of foodborne pathogenic infections (Galie et al., 2018). *V. harveyi* is a Gram-negative bacterium in a free-living state in marine environmental condition. It is commonly present as a commensal microflora, but it also can cause significant harm as a pathogenic to shrimp and other seafoods (Yang and Defoirdt, 2015). *V. parahaemolyticus* establishes colonization on shellfish surface, and has been recognized as the primary causative of seafood-related gastroenteritis (Lee et al., 2023). The human-pathogenic strains and aquatic animal-pathogenic strains are distinct strains, and that some *Vibrio* strains are not pathogenic to human beings. Hence, novel antimicrobial agents are required that can inhibit growth of planktonic and biofilms as well as virulence factors.

Notably, various antimicrobial agents are replete with halogen atoms. The halogen atoms play a substantial and increasingly imperative role in the context of electronegative elements that possess accessible ion pairs and can form complexes with hydrogen bond acceptors. The characterization of this behavior has been elucidated based on molecular level of electrostatic impending surfaces (Cavallo et al., 2016). In addition, extensive research has examined the impact of halogenation, specifically bromination, chlorination, and iodination, on antimicrobial activity (Faleye et al., 2024). The halogenation process is a valuable approach for manipulating the characteristics of biologically active compounds, particularly antimicrobial agents or example, boronic acids are intrinsically reactive yet stable and have low toxicity (Jia et al., 2019; Faleye et al., 2024). Boronic acids are bioisosteres of carboxylic acids because they have the same period as carbon. Boronic acids, which are saccharide binders, may assist in investigating biological systems and finding diabetes pathogenesis metabolites (James et al., 2006). In addition, boronic acids, which are mild Lewis acids, are essential for organic synthesis and cross-coupling because of their stability and ease of use (Tornesello et al., 2020) and have been used as a functional group for anticancer, antiviral, and antimicrobial activities (Trippier and McGuigan, 2010). Therefore, it was hypothesized that various halogenated acids including boronic acids would have antimicrobial and antibiofilm activity against *Vibrio* species.

33 halogenated acids were tested against *V. harveyi* and *V. parahaemolyticus* and two active compounds, 3,5-diiodo-2-methoxyphenylboronic acid (DIMPBA) and 2-fluoro-5-iodophenylboronic acid (FIPBA), eradicated the biofilms of *V. parahaemolyticus.* In addition, these active compounds can hinder the development of biofilm-associated virulence factors, including swimming and swarming motility, aggregation, hydrophobicity, protease activity, and indole synthesis. Moreover, the food preservation capabilities of the two active halogenated acid derivatives were evaluated by modeling a marine food product after prawns and shrimp.

## 2 Materials and methods

### 2.1 Bacterial strains and chemicals

The *V. parahaemolyticus* ATCC 17802 and *V. harveyi* ATCC 14126 procured from American collection culture center (Manassas, USA) were used. Two bacterial strains were used for experiments in marine Luria–Bertani media (mLB) with 3% NaCl (w/v). All the experimental procedures were performed at 30°C in mLB solid agar plates and liquid media. Halogenated acid and its thirty-three derivatives were purchased from Combi-Blocks, Inc. (San Diego, USA), and Sigma–Aldrich (St. Louis, USA) (**Table 1**): boronic acid, 3,5-diiodo-2-methoxyphenylboronic acid, 2-fluoro-5-iodophenylboronic acid, 4-iodo phenylboronic acid, 2-amino-5-iodobenzoic acid, 2-chloro-5-iodobenzoic acid, 4-chloro-2-iodobenzoic acid, 4-chloro-3-iodobenzoic acid, 5-chloro-2-iodobenzoic acid, 2,4-difluoro-5-iodobenzoic acid, 2,5-diiodo benzoic acid, 2-fluoro- 4 iodo benzoic acid, 2-fluoro 6-iodo benzoic acid, 3-fluoro-4-iodobenzoic acid, 5-fluoro-2-iodobenzoic acid, 3-hydroxyl-4-iodobenzic acid, 2-iodobenzoic acid, 3-iodobenzoic acid, 4-iodobenzoic acid, 2-iodo-5-methoxybenzoic acid, 3-iodo-4-methoxybenzoic acid, 2-iodo-3-methylbenzoic acid, 2-iodo-5-methylbenzoic acid, 3-iodo-2-methylbenzoic acid, 3-iodo-4-methylbenzoic acid, 4-iodo-3-methylbenzoic acid, 5-iodo-2-methylbenzoic acid, 4- iodo phenoxyacetic acid, 2-iodo phenylacetic acid, 4-iodo phenylacetic acid, 3- (4-iodophenyl) propionic acid, 3-iodophthalic acid, 3-iodo propionic acid, and 2,3,5 –triiodobenzoc acid. The halogenated acids were diluted in dimethyl sulfoxide (DMSO). A negative control was also used, consisting of DMSO (0.1% v/v). For the biotic surface test, frozen squid (*Todarodespacificus*) and shrimp (*Penaeus vannamei*) were obtained from Gyeongsan, South Korea, and refrigerated at -20°C for further use.

**Table 1:** This study observed the minimum inhibitory concentration (MIC) and planktonic cell growth of 34 halogenated acids against *V. harveyi* and *V. parahaemolyticus*.

No	Compound name	Structure	*V. parahaemolyticus*			*V. harveyi*		
			MIC (µg/mL)	Growth inhibition (%)		MIC (µg/mL)	Growth inhibition (%)	
				50 µg/mL	100 µg/mL		50 µg/mL	100 µg/mL
1.	Boronic acid	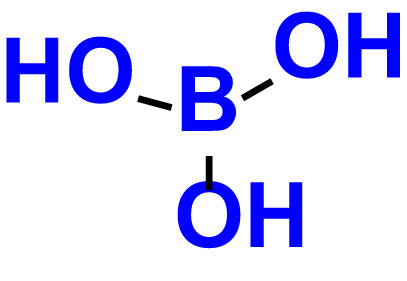	>500	76.7	82.6	>500	106.0	113.5
2.	3,5-Diiodo-2-methoxyphenylboronic acid (DIMPBA)	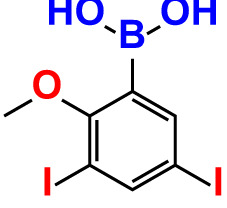	100	30.3	0.8	200	81.7	16.1
3.	2-Fluoro-5-iodophenylboronic acid (FIPBA)	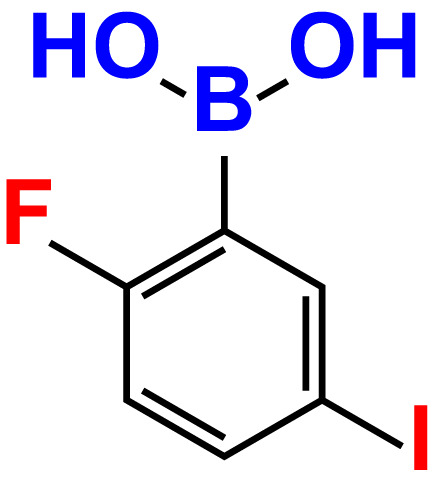	100	24.1	16.0	150	107	70.3
4.	4-Iodophenylboronic acid	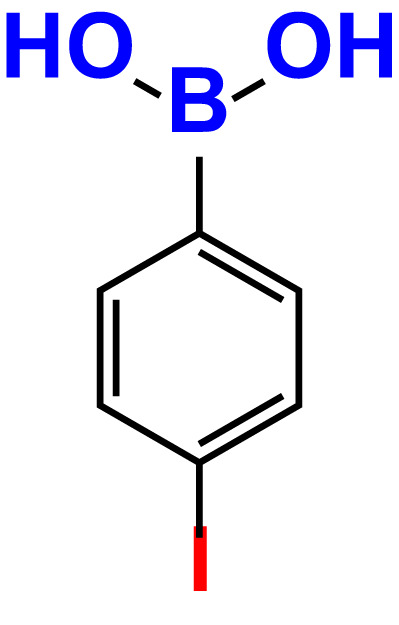	200	62.1	12.7	>500	99.8	67.6
5.	2-Amino-5-iodobenzoic acid	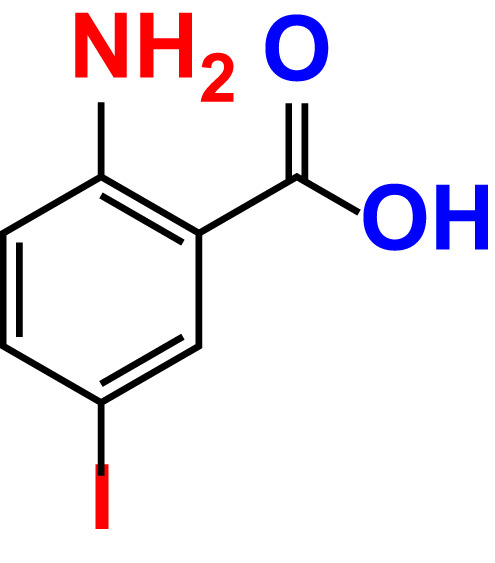	>500	103.5	108.7	>500	103.4	122.5
6.	2-Chloro-5-iodobenzoic acid	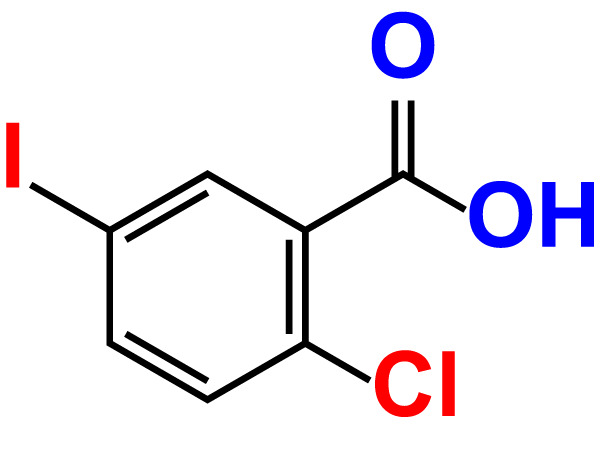	>500	86.9	96.7	>500	97.3	103.7
7.	4-Chloro-2-iodobenzoic acid	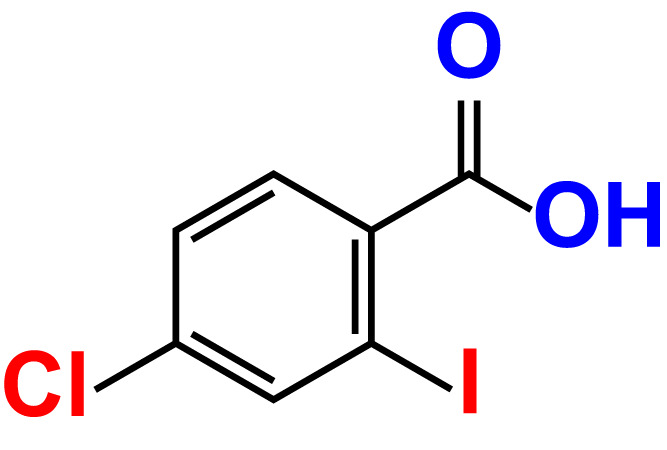	>500	97.4	101.4	>500	97.4	109.8
8.	4-Chloro-3-iodobenzoic acid	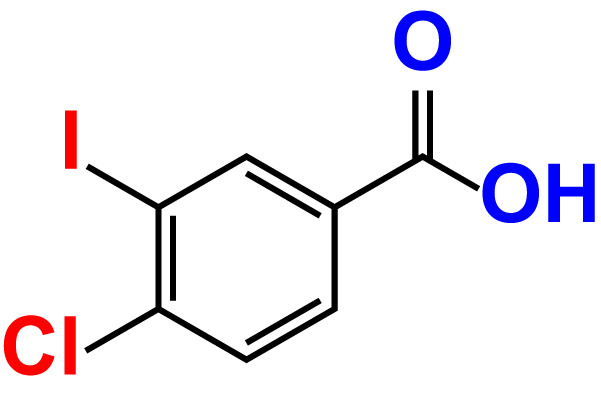	>500	95.9	90.1	>500	85.4	110.0
9.	5-Chloro-2-iodobenzoic acid	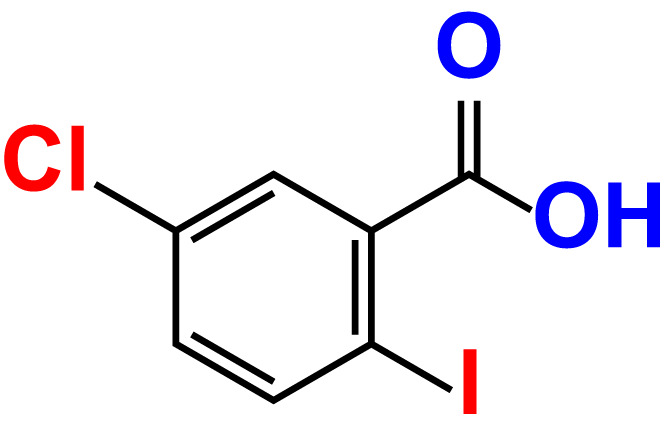	>500	81.2	92.4	>500	92.3	112.6
10.	2,4-Difluoro-5-iodobenzoic acid	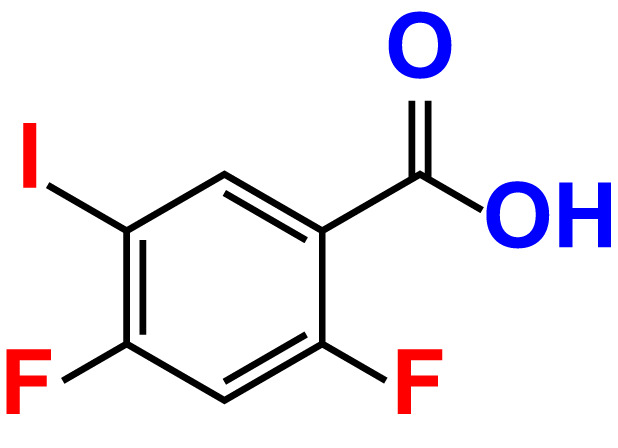	>500	94.2	101.5	>500	95.0	115.7
11.	2,5-Diiodobenzoic acid	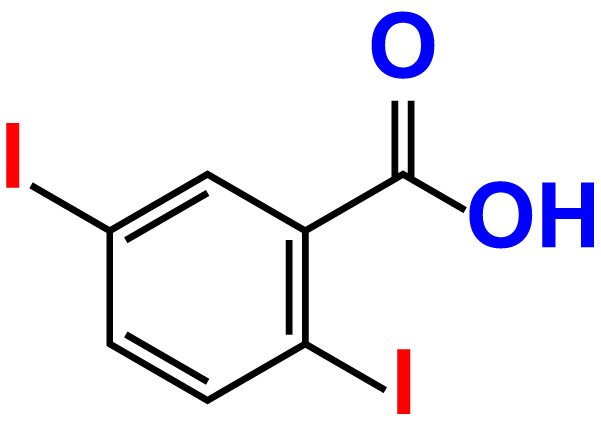	>500	100.2	99.1	>500	94.3	102.8
12.	2-Fluoro-4-iodobenzoic acid	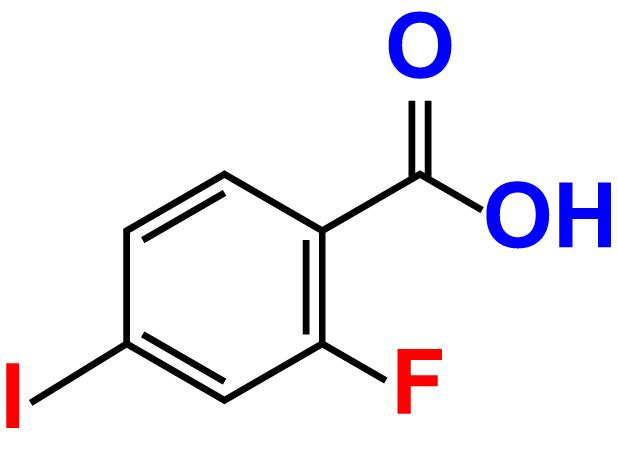	>500	89.4	93.6	>500	93.7	113.9
13.	2-Fluoro-6-iodobenzoic acid	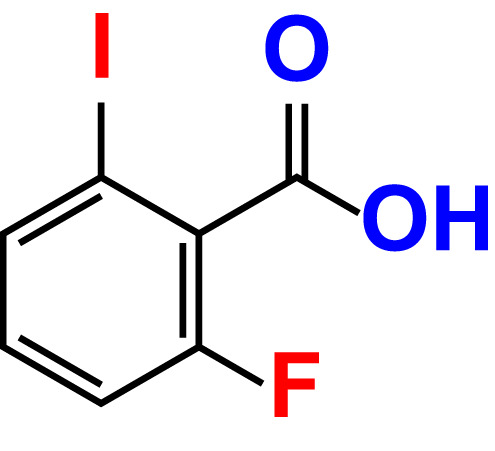	>500	86.5	90.5	>500	89.5	105.4
14.	3-Fluoro-4-iodobenzoic acid	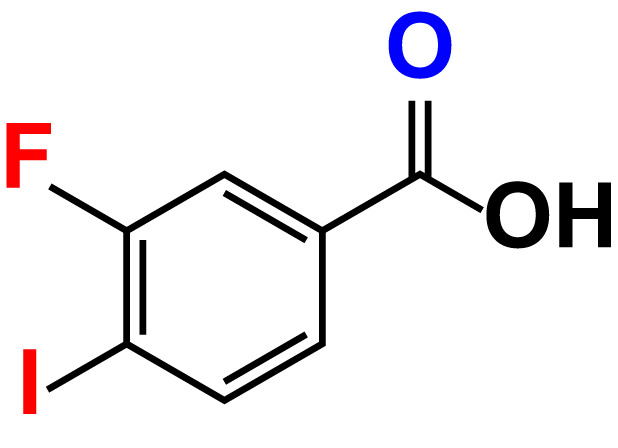	>500	90.6	92.9	>500	93.4	107.2
15.	5-Fluoro-2-iodobenzoic acid	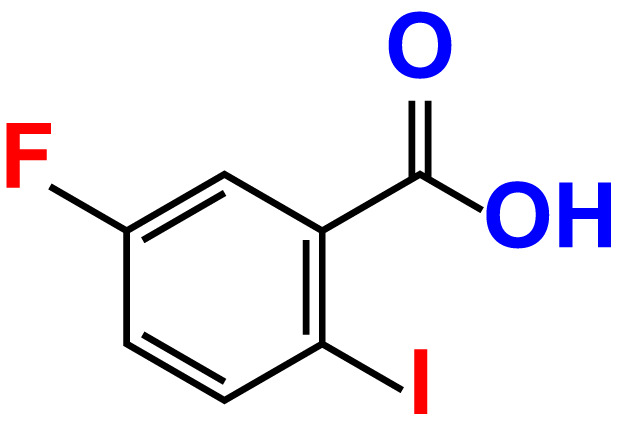	>500	84.9	90.1	>500	88.7	101.4
16.	3-Hydroxy-4-iodobenzoic acid	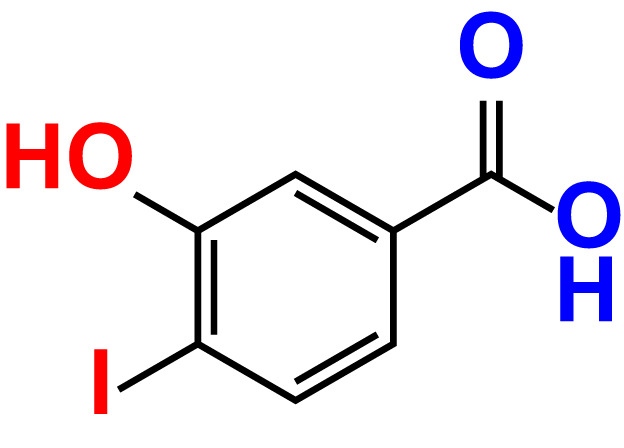	>500	83.0	86.1	>500	89.4	112.4
17.	2-Iodobenzoic acid	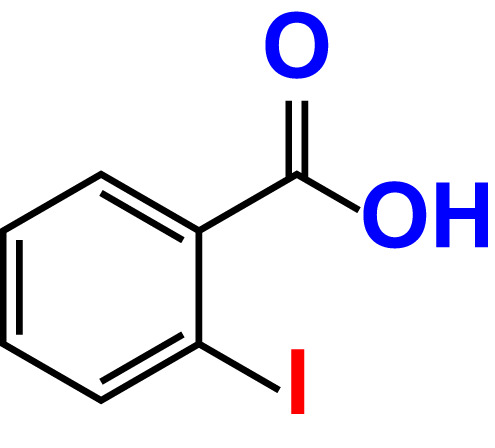	>500	84.1	89.3	>500	92.6	107.7
18.	3-Iodobenzoic acid	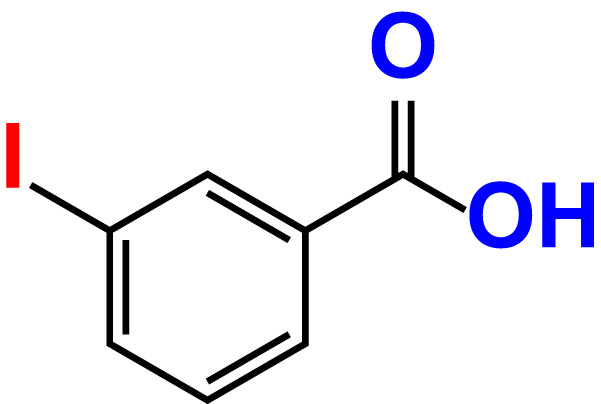	>500	97.3	98.5	>500	90.4	106.9
19.	4-Iodobenzoic acid	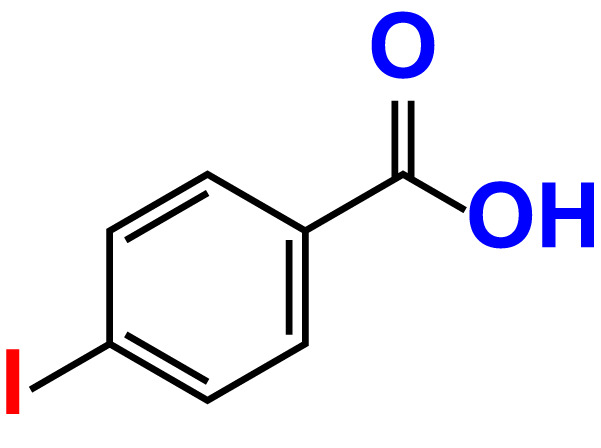	>500	83.4	84.2	>500	87.8	102.7
20.	2-Iodo-5-methoxybenzoic acid	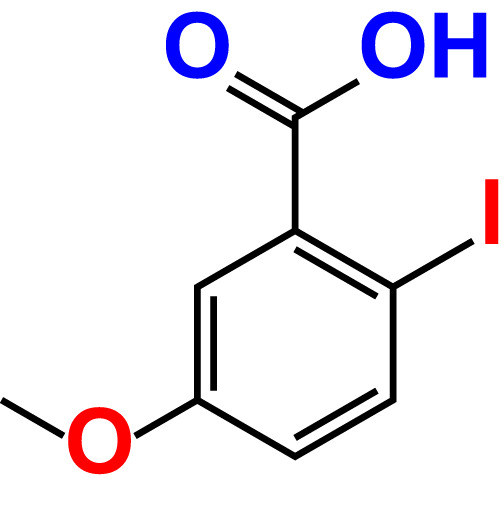	>500	81.0	88.7	>500	87.7	107.4
21.	3-Iodo-4-methoxybenzoic acid	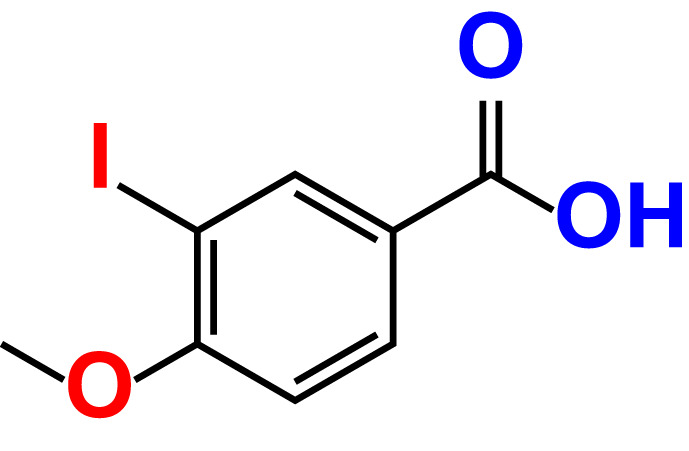	>500	88.2	86.5	>500	94.8	110.1
22.	2-Iodo-3-methylbenzoic acid	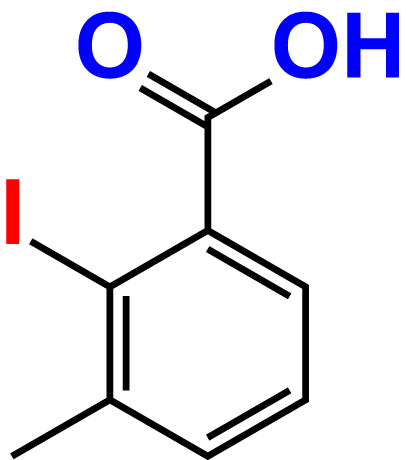	>500	84.7	93.1	>500	103.9	126.8
23.	2-Iodo-5-methylbenzoic acid	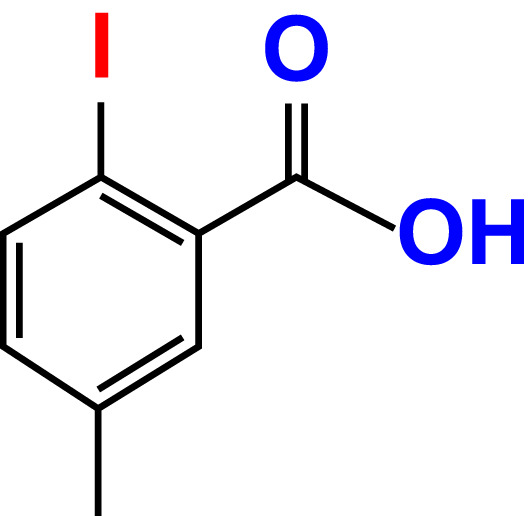	>500	81.2	84.3	>500	96.8	119.2
24.	3-Iodo-2-methylbenzoic acid	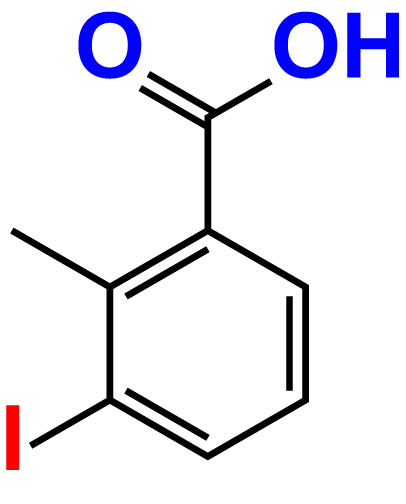	>500	85.4	102.5	>500	101.8	122.4
25.	3-Iodo-4-methylbenzoic acid	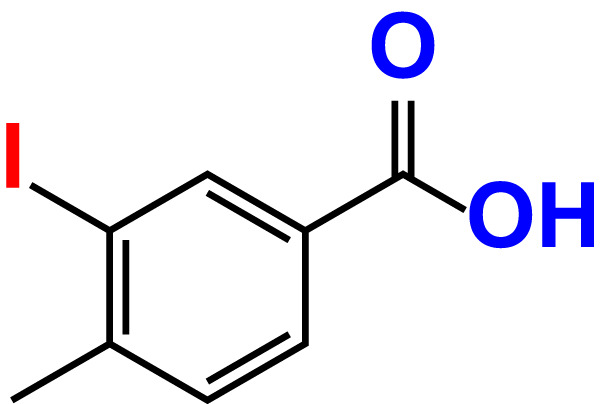	>500	91.5	81.5	>500	103.3	111.4
26.	4-Iodo-3-methylbenzoic acid	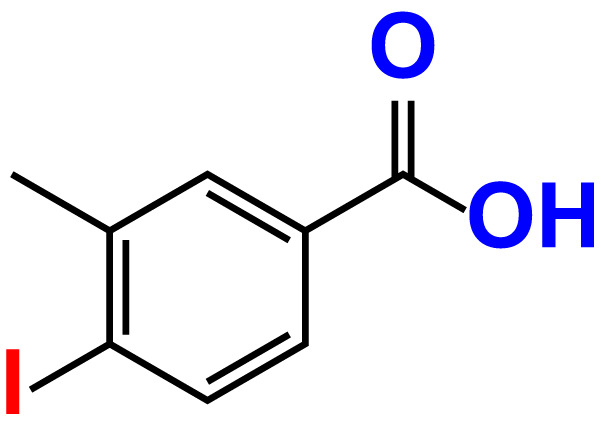	>500	82.6	81.2	>500	107.7	107.6
27.	5-Iodo-2-methylbenzoic acid	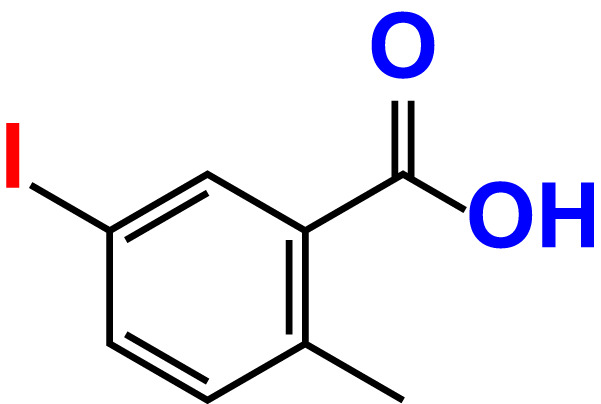	>500	81.5	90.9	>500	104.0	118.9
28.	4-Iodophenoxyacetic acid	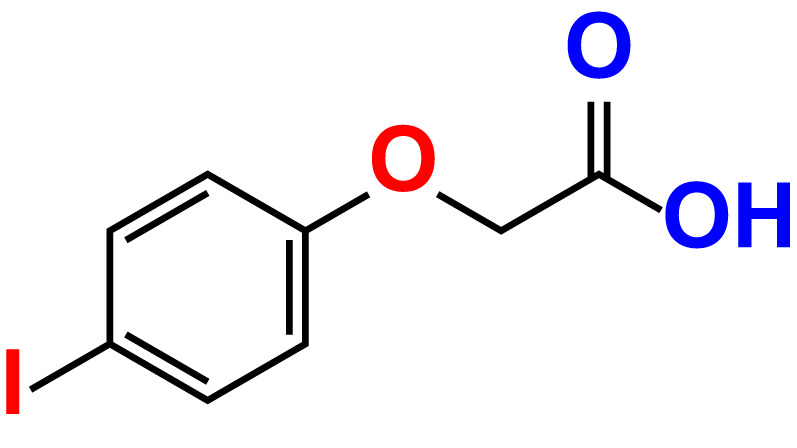	>500	81.2	87.9	>500	96.2	113.9
29.	2-Iodophenylacetic acid	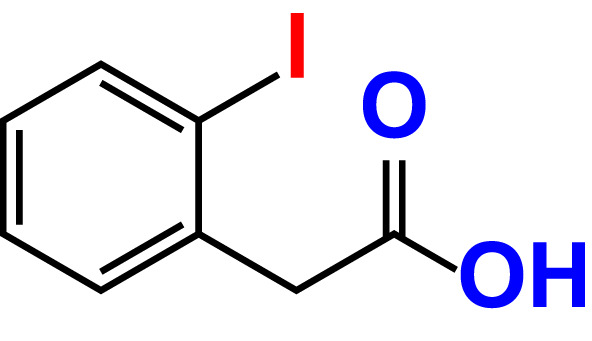	>500	82.2	86.9	>500	98.5	118.1
30.	4-Iodophenylacetic acid	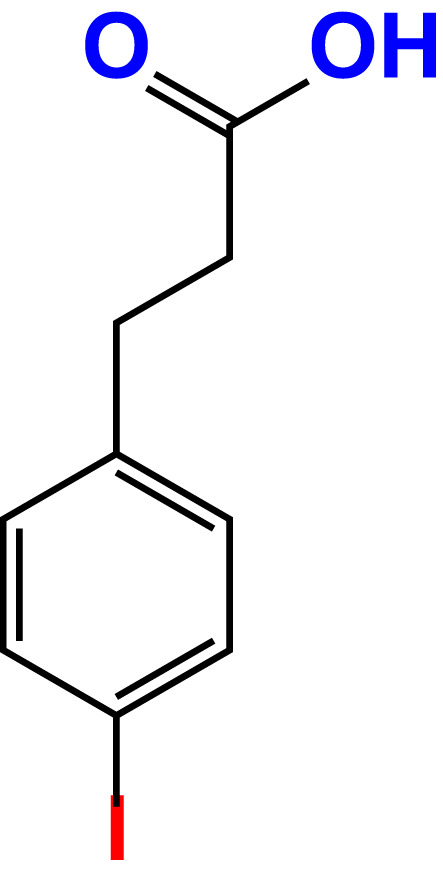	>500	87.0	81.8	>500	96.4	113.8
31.	3-(4-Iodophenyl)propionic acid	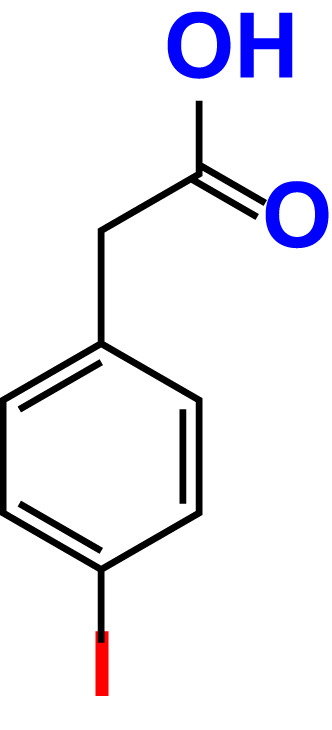	>500	89.7	97.2	>500	93.9	101.1
32.	3-Iodophthalic acid	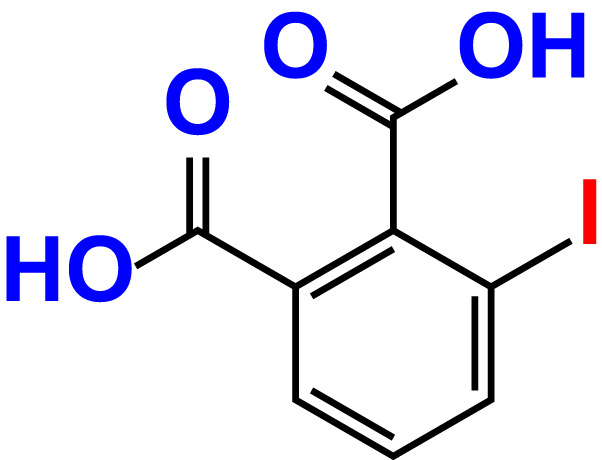	>500	85.9	89.2	>500	117.2	127.1
33.	3-Iodopropionic acid	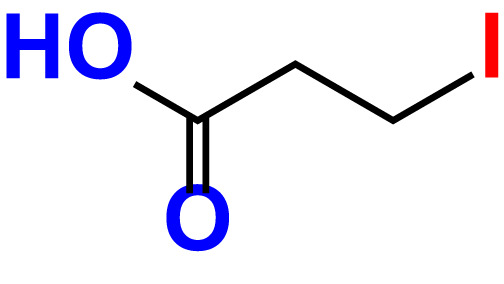	>500	82.2	86.9	>500	106.3	122.5
34.	2,3,5-Triiodobenzoic acid	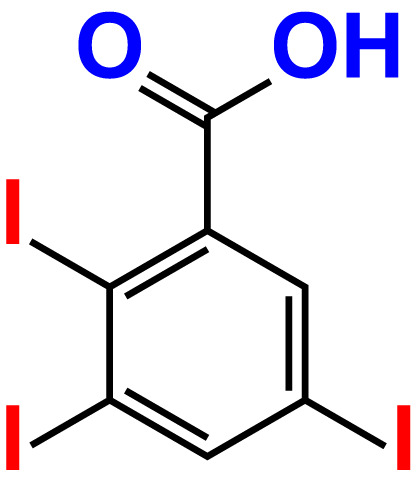	>500	105.1	110.2	100	109.2	127.5

The boronic acid and the two active hits compounds were highlighted in blue font.

### 2.2 Bacterial cell growth and minimum inhibitory concentration

The overnight-grown *Vibrio* culture was reinoculated at a part of 1:100 (~6.4×10^7^ CFU) in liquid mLB media and maintained in an incubator under stationary conditions at 30°C for 24 h. The cell OD at 620 nm was monitored for 24 h using a EX Multiskan microplate reader manufactured by Thermo Fisher Scientific in the United States. A slight modification of the microdilution broth technique was used to measure the MICs of *V. parahaemolyticus* and *V. harveyi*. Briefly, both strains were treated with different concentrations (10, 20, 50, 100, 200, 400, and 500 μg/mL) of DIMPBA and FIPBA on 96-well plates after diluting 1:100 (OD 0.1) in mLB overnight. The MIC is the concentration of treatment compound at which there is no discernible growth during static incubation for 24 h at 30°C (Sathiyamoorthi et al., 2021). The data represents the average across at minimum three distinct independent cultures.

### 2.3 Assessment of biofilm inhibition assay

The antibiofilm activity of halogenated acids against *V. parahaemolyticus* was considered using a modified version of the crystal violet method (Faleye et al., 2021). *V. parahaemolyticus* that had been grown overnight was prepared using mLB liquid media at a dilution of 1:100. Subsequently, the culture was shifted to a 96-well plates with a volume of 300 µL in each well (SPL Life Sciences, Korea). The plates were kept under stagnant conditions for 24 h at 30°C in the presence of halogenated acids at 10, 20, 50, 75, 100, 125, 150, and 200 µg/mL. The sample was washed thrice to eliminate non-adherent cells. Each well was stained with 0.1% crystal violet for 20 min followed by 95% ethanol after thoroughly rinsing with double distill water. A Multiskan EX microplate reader (Thermo Fisher Scientific) assessed biofilm cells at 570 nm. The average of six replicated 96-wells were used to conclude the quantity of biofilm formation.

### 2.4 Visualization of *V. Parahaemolyticus* in live cell imaging microscopy

The microscopic techniques were employed to observe the effect of potent compounds (Kim et al., 2022). *V. parahaemolyticus* was cultured overnight and then mixed with 1:100 in mLB liquid medium for live cell imaging. The 96 well-plates were kept at 30°C, with the addition of halogenated acids at concentrations of 10 to 200 µg/mL. The incubation was carried out in stationary positions for 24 h. The previously used liquid media were removed and rinsed with a phosphate-buffered saline (PBS) three times to facilitate the visualization of biofilms. The iRiS Digital Cell Biofilms Imaging System, which was developed by Logos BioSystems (Anyang, Korea), was used to examine the biofilms at different magnifications. Further, biofilm images were processed using the ImageJ imaging program to generate colored 3-D representations.

### 2.5 Bacterial swim and swarm motility

The swimming motility of *V. parahaemolyticus* was evaluated by cultivating the bacterium on mLB semi-solid plates supplemented with 0.3% of agar. Various concentrations (10, 20, 50, 100, and 200 µg/mL) of halogenated acids were incorporated into the culture medium. The one µL of 24 h grown *V. parahaemolyticus* was meticulously spotted at the epicenter of the semi-solid agar plate and kept in an upright orientation. The swarming motility was promoted by fortifying the mLB plates with a 0.5% agarose additive. Subsequently, the plates were sealed and subjected to an inverted orientation for 24 h at a temperature of 30°C (Sathiyamoorthi et al., 2021).

### 2.6 Agglutination assay using *Saccharomyces cerevisiae*

The agglutination was evaluated using halogenated acid derivatives, following the methodology (Sethupathy et al., 2020). The 24 h cultures of *V. parahaemolyticus* were mixed at a ratio of 1:100 dilution. These diluted cultures were treated with halogenated acid derivatives at 30°C for 24 h while being agitated at 250 rpm. The optical density of the cells was adjusted 0.5. Subsequently, 400 µL of the culture was transferred into tubes containing volume of 1500 µL of PBS and 500 µL of a 2% concentration (2 g of yeast *S. cerevisiae* mixed within 10 mL of PBS solution) of yeast *S. cerevisiae* (Sigma–Aldrich, USA). Afterwards brief vortexing of the mixture for a duration of 5 sec, the initial OD was determined at 600 nm (Optizen spectro 2120UV, Korea). Incubation was continued at room temperature for 25 min. A 100 µL volume of clear liquid after vigorous vortex was moved into 96-well plates, and the OD measured at 600 nm was determined. The agglutination percentage was calculated by using the following formula: 100 × (1 − (OD_600before_/(OD_600after_)).

### 2.7 Hydrophobicity assay

Based on previous work (Sathiyamoorthi et al., 2021), the effects of halogenated acid on cell surface hydrophobicity was investigated. The culture grown overnight was diluted at a ratio of 1:100 dilution. The diluted culture was mixed with halogenated acids at 0, 10, 20, 50, 100, and 200 µg/mL. Subsequently, the culture was kept at 30°C with a rotational speed of 250 rpm for the duration of 24 h. Following the cultivation process, 1 mL from the cultured sample was centrifuged at 13000×g for 20 min. The cell pellets were washed and resuspended into the 4 mL of sterile PBS. The optical density of the bacterial cells at 600 nm was adjusted to approximately 0.5, denoted as Ao. An additional 1 mL of hexadecane was added to the mixture was vortexed vigorously for 1 min to separate the two phases of layers. Consequently, the aqueous bottom phase (1 mL) was extracted meticulously, and the ultimate OD at 600 nm (Ai) was determined (Optizen 2120UV, Korea). The formula calculated: Hydrophobicity (H) % = (Ao − Ai)/Ao × 100. The statistical values illustrate the mean standards deviation of six separate cultural groups.

### 2.8 Protease assay

The bacterial protease production was quantified using the methodology described previously (Faleye et al., 2021). *V. parahaemolyticus* was mixed with halogenated acid at 10, 20, 50, 100, and 200 µg/mL and the treatment were conducted for 24 h at 30°C and 250 rpm. After a 24 h incubation period, the microtube underwent centrifugation at a force of 13,000 × for 10 min. Subsequently, 75 µL of the aqueous phase was treated with a solution containing 2% weight/volume azocasein, with a volume of 125 µL and stored at 37°C for 25 min. As a result, trichloroacetic acid of 10% prepared, and 600 µL was added to the mixture to inhibit the proteolytic activity. Subsequently, the tubes were stored at −20°C for 20 min. Furthermore, a NaOH solution (1 M, 700 µL) was incorporated into the suspension. The absorbance at 440 nm of the mixture was measured and the mean values were measured from a sample of six independent cultures.

### 2.9 Visualization under scanning electron microscopy

SEM was used to perceive the formation of *V. parahaemolyticus* biofilms on nitrocellulose membranes measuring 0.5 × 0.5 cm. This experimental setup was previously described (Sathiyamoorthi et al., 2021). The biofilm development was examined in the presence or absence of halogenated acid derivatives (100 µg/mL). The biofilms developed under stagnant conditions at 30°C for 24 h. As a result, cells adhering cellulose nylon membrane were fixed using a solution containing 2% formaldehyde and 2.5% glutaraldehyde overnight at 4°C. Subsequently, the samples underwent dehydration using ethanol (30%, 50%, 70%, 80%, 95%, and 100%) at different concentrations for 10 min each. The drying method was used, followed by sputter coating the specimens with gold or platinum. Finally, the specimens were scanned by (FESEM, Hitachi S-4200, Hitachi, Japan) operated at 10 kV at various magnifications.

### 2.10 Indole investigate with different pH

Indole production was evaluated in the occurrence of halogenated acid derivatives at various pH (Sathiyamoorthi et al., 2023a). In summary, the culture that had grown overnight was dilute using a 1:100 dilution with mLB. Subsequently, the diluted culture was exposed to halogenated acid at 10, 20, 50, 100, and 200 µg/mL for 24 h. The treatment was conducted at 30°C with an agitation speed of 250 rpm. The liquid media was prepared at a neutral pH of 7. Subsequently, the pH of the media was maintained to pH 5 using a 35% HCl solution and to pH 9 using a 5 N NaOH solution. After a 10 h of incubation period, a 1 mL sample of the cell culture was centrifuged at a force of 11,000 rpm the acceleration due to gravity for 10 min. Subsequently, a 1000 µL portion of the supernatant was combined with 300 µL of Kovac’s solution, consisting of (10 g, *p*-dimethyl amino benzaldehyde, 50 mL of 35% HCl, and 150 mL of amyl alcohol). Then the reaction mixture was permitted to proceed for 2 min at ambient condition. The uppermost stratum of 50 µL was transferred meticulously into a cuvette containing 950 µL of the HCl-amyl alcohol. The absorbance of indole was conducted at 540 nm. The provided data corresponds to the average values obtained from six distinct cultures.

### 2.11 Dispersal assay

The biofilm dispersal assay was conducted to assess the efficacy of halogenated acids in disrupting cells of *V. parahaemolyticus*. After 24 h of biofilm formation at 30°C, the planktonic cells were subjected for rinsing using PBS with a pH value of 7.4. Subsequently, newly prepared mLB liquid media with different concentrations of halogenated acids (10 to 200 µg/mL) were added to the plates and incubated under stationary position at 30°C for an additional 24 h. The biofilms were subsequently rinsed with water to eliminate any non-adherent bacterial cells. Subsequently, the biofilm was treated with crystal violet solution for 25 min and rinsed with water before being exposed to 95% ethanol. The absorbance reading was taken at 570 nm. The results are presented as the average of 12 replicated wells.

### 2.12 Investigation of the biotic surfaces of squid and shrimp

The efficacy of halogenated acids in inhibiting the bacterial growth of *V. parahaemolyticus* and the formation of biofilms on seafood surfaces was demonstrated as previously reported (Faleye et al., 2021). The squid specimen underwent a meticulous preparation process involving carefully separating the main body and mantle from the hood and tentacles. The squid body was divided into 1.5 × 1.5 × 0.5 cm sections using a sterile scalpel within a sterile Petri dish. The squid were washed with 3% of sodium hypochlorite, and rinsed thrice with double distilled water. Then fragments were placed within a safety chamber cabinet for one hour. The samples were then allocated into distinct cohorts, and the respective interventions were administered: the surface of the squid was subjected to treatment with *V. parahaemolyticus* at 20 µg/mL of the potent compounds. The samples were initially contaminated with a concentration of 5.6 × 10^4^ CFU per milliliter and were then incubated for 24 h at a temperature of 30°C under static conditions. The squid samples were prepared for the SEM examination, as reported previously (Sathiyamoorthi et al., 2021).

Cooked shrimp specimens were employed to assess the efficacy of the halogenated acid derivatives in prolonging the seafood shelf life (Sathiyamoorthi et al., 2023b). The defrosted shrimp samples, weighing 1.0 g, were washed with distilled water. Subsequently, the samples were subjected to UV irradiation for approximately 30 min in a safety cabinet (JSCB-1200SB; Korea). This procedure aimed to eliminate the residual microorganisms present, with 15 min of UV exposure on the front and backsides of the samples. The shrimp specimens were infected with *V. parahaemolyticus* by immersion in a bacterial mixture for five min, resulting in a 6 log CFU/g. Subsequently, 10 min were allocated for the specimens to undergo air-drying within the confines of the biosafety clean chamber cabinet. After inoculation, the samples were partitioned and immersed in solutions containing the halogenated acids at concentrations of 10 to 200 μg/mL for 15 min. Then the shrimp specimens were placed in sterile plastic bags and kept at 4°C for a duration of five days. The specimens were obtained at consistent time intervals and subjected to vigorous vortexing to dislodge the cells. The occurrence of *V. parahaemolyticus* on the shrimp was determined using colony-forming units (CFU).

### 2.13 ADME profile

ADME software was used to analyze the drug-like qualities of DIMPBA and FIPBA (Daina et al., 2017). Online web servers, e.g., Molinspiration (https://www.molinspiration.com/), PreADMET (https://preadmet.qsarhub.com/), and GUSAR (http://www.way2drug.com/gusar/) were performed on 16 August 2023. According to Lipinski’s rules of five, an orally active medication should possess certain characteristics. These include a molecular weight of ≤ 500 g/mol, log P of ≤ 5, ≤ 5 hydrogen bond-donating atoms, no more than 10 hydrogen-bond-accepting atoms, and an octanol-water partition coefficient of ≤ 140 Å2 or less (Lipinski et al., 1997).

### 2.14 Statistical evaluation

The experimental design encompassed three distinct cultures and six replicates. The data presented are described as mean values ± standard deviation (SD) designated by the ± symbol. The mean significance was assessed using a Student’s t-test with an impact threshold of p < 0.05.

## 3 Results

### 3.1 Biofilm formation and growth by two *Vibrio* species was inhibited by halogenated acids

The MIC test was initially evaluated to assess 34 halogenated acids for their antibacterial activity against two strains of *Vibrio* species. According to **Table 1**, *V. harveyi* and *V. parahaemolyticus* showed a similar tendency in the antimicrobial efficacy of the 34 halogenated acids. Particularly, the MICs of DIMPBA and FIPBA were 100 μg/mL, whereas 4-iodophenylboronic acid showed a MIC of 200 μg/mL. In contrast, other 31 halogenated acid derivatives did not exhibit any influence on the cell growth, even at concentrations exceeding 500 μg/mL, as indicated in **Table 1**.

**Figure 1:**
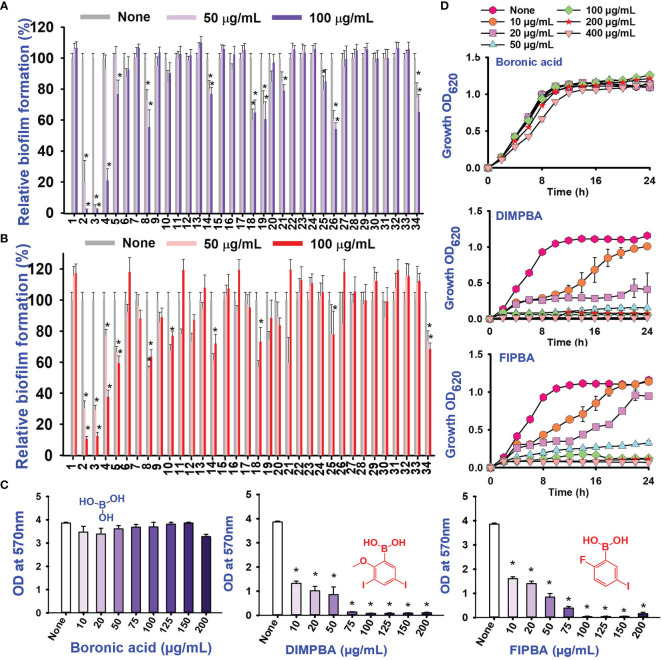
Inhibitory effects of 34 halogenated acids on biofilm formation by *V. parahaemolyticus* **(A)** and *V. harveyi* **(B)**. The biofilm inhibition activity against *V. parahaemolyticus* by halogenated acid and its two active compounds 3,5-diiodo-2-methoxyphenylboronic (DIMPBA) and 2-fluoro-5-iodophenylboronic acid (FIPBA) **(C)**. The growth curve of planktonic *V. parahaemolyticus* cells in the presence of DIMPBA and FIPBA halogenated acids **(D)**. * indicates p < 0.05.

Also, thirty-four halogenated derivatives were screened at 50 and 100 μg/mL to assess their antibiofilm efficacy against *V. parahaemolyticus* and *V. harveyi* were significantly inhibited the biofilm formation. On the other hand, the backbone of halogenated acid (boronic acid) did not depict the antibiofilm activity (**Table 1**; **Figures 1A, B**). Furthermore, extensive biofilm assay showed that two boronic acid derivatives (DIMPBA and FIPBA) exhibited a dose-dependent decrease in biofilm formation in *V. parahaemolyticus* at 10, 20, 75, 100, 125, 150, and 200 μg/mL, demonstrating the antibacterial and antibiofilm impact on bacterial growth (**Figure 1C**). Also, the halogenated acids, i.e., DIMPBA and FIPBA, were used to monitor the bacterial growth curve for 24 h. The boronic acid didn’t exhibit notable planktonic cell growth inhibition. In contrast, DIMPBA and FIPBA showed complete cell growth inhibition at 100 µg/mL (**Figure 1D**).

### 3.2 Boronic acid derivatives inhibited biofilm formation, swimming and swarming motilities, and SEM

**Figure 2:**
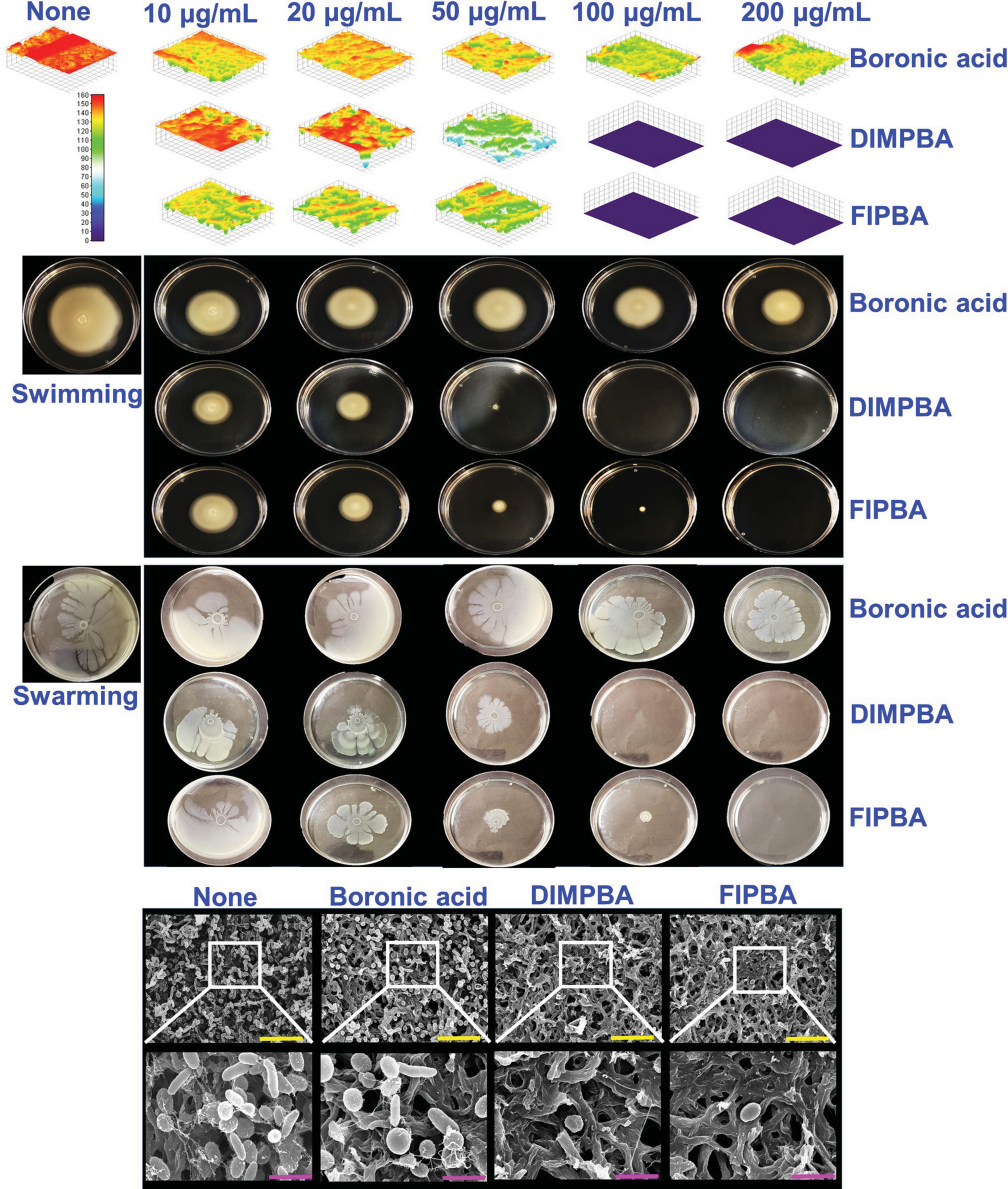
Inhibition of *V. parahaemolyticus* biofilms by active halogenated acids observed through a microscopic evaluation **(A)**. Impact of halogenated acids on the bacterial surface motility of *V. parahaemolyticus*. Swimming motility **(B)** and swarming motility **(C)**. SEM **(D)** visualized the biofilm cells subjected to boronic acid, DIMPBA, and FIPBA (100 μg/mL). The yellow and pink bars correspond to measurements of 6 and 1.5 µm, respectively.

The live cell imaging and motilities assays showed that boronic acid derivatives had an antibiofilm impact against *V. parahaemolyticus*. The use of a 3D cell imaging showed that the presence of DIMPBA and FIPBA at 100 μg/mL had a considerable decrease in biofilm thickness when compared with the untreated control condition (**Figure 2A**).

Flagella showed a crucial role in facilitating the movement and association of microorganisms and expediting biofilm formation (Lemon Katherine et al., 2007). The effect of boronic acid derivatives on motility was measured in semi-solid agar plates and results revealed a noticeable difference in the swimming and swarming motilities. The motility assays of swimming and swarming were observed to be reduced dose-dependently by two boronic acid derivatives compared to boronic acid. In particular, at 100 μg/mL, DIMPBA and FIPBA exhibited the complete inhibition of motility assays. Specifically, the swimming motility of the subject decreased by 95.2% and 82.0% when exposed to 50 μg/mL DIMPBA and FIPBA, respectively.

Similarly, the swarming motility decreased by 51% and 97.1% when exposed to 50 μg/mL DIMPBA and FIPBA (**Supplementary Figure 1**), respectively (**Figures 2B, C**). SEM showed that the untreated control group exhibited adhesion between cells, forming a biofilm. This biofilm was predominantly covered by a substance resembling a mucous membrane. Within this matrix, the bacteria displayed their typical rod-shaped morphology, characteristic of *V. parahaemolyticus* cells. In contrast, the bacterial cells treated with DIMPBA and FIPBA at 100 μg/mL showed a noticeable decrease in biofilm formation. This reduction was evident by the decrease in bacterial cells and extracellular material (**Figure 2D**).

### 3.3 Halogenated acids decreased the formation of fimbria, hydrophobicity, protease, and indole production

**Figure 3:**
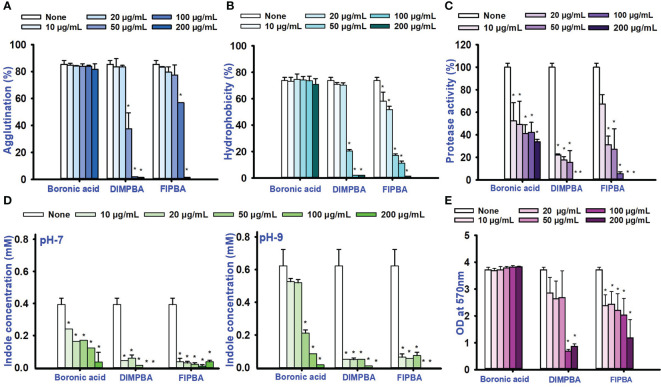
Activity of fimbriae and the influence of halogenated acids **(A)**, hydrophobicity of the cell surface **(B)**, protease assay **(C)**, and the production of indole was examined under conditions of pH 7 and pH 9 **(D)**. The matured biofilm eradication of biofilm formation for boronic acid, DIMPBA, and FIPBA **(E)**. The asterisk (*) denotes statistical significance at a significance level of p < 0.05.

This study investigated the impacts of fimbrial by exploring the role of yeast cell agglutination. The two boronic acid derivatives, DIMPBA and FIPBA, exhibited a decrease in fimbria activity at a concentration of 50 μg/mL, and the reduction observed was 37.7% and 77.3% for DIMPBA and FIPBA, respectively. Furthermore, at 100 μg/mL, these two derivatives showed total suppression of fimbria activity (**Figure 3A**). The hydrophobicity is directly associated with the initial adhesion and subsequent biofilm formation. On the other hand, a reduction in hydrophobicity is detrimental to the biofilm formation (Chang et al., 2012). According to the literature, the established rates of hydrophobicity can be categorized as follows: a hydrophobicity > 50% is measured highly hydrophobic in nature; a value of 20–50% is classified as moderate hydrophobic; a value < 20% is categorized as non-hydrophobic (Saeed and Heczko, 2007). The experimental findings indicate that the control group, boronic acid, exhibited a relative hydrophobicity of 73.4%. In contrast, the halogenated derivatives DIMPBA and FIPBA displayed significantly lower hydrophobicity, ranging from 98% to 88.8% at 100 μg/mL, and these derivatives exhibited hydrophilic at this concentration and complete inhibition at higher concentrations (**Figure 3B**). Also, the protease activity was significantly decreased in a dose-dependent manner by DIMPBA and FIPBA, with 100% and 94.4% reductions, respectively, at 100 μg/mL. (**Figure 3C**).

Bacterial cell signaling indole plays significant role in the competition for space and nourishment within environmental conditions consisting of diverse bacteria (Lee and Lee, 2010). The concentration of intracellular and extracellular indole affects the physiology of *Vibrio* species (Mueller et al., 2009). Similarly, according to these findings, the indole levels were higher at pH 9 compare than at pH 5 and 7. Hence, pH is crucial for synthesizing indole because it is suppressed by acidic pH condition but more favored for neutral condition and alkaline conditions. The production of indole was significantly reduced in a dose-dependent way by DIMPBA and FIPBA (**Figure 3D**; **Supplementary Figure 2**). Furthermore, the dispersal assay suggests that halogenated acids have a dose-dependent effect on disrupting the biofilm formation. The active compound DIMPBA exhibited a significant reduction in mature biofilms, with an 81.5% and 45.3% reduction at 100 μg/mL for DIMPBA and FIPBA, respectively. These results demonstrated that two boronic acid acids have the efficacy in inhibiting the biofilm formations or eliminating established biofilms (**Figure 3E**).

### 3.4 Halogenated acids inhibited biofilm formation on the squid and shrimp surfaces

**Figure 4:**
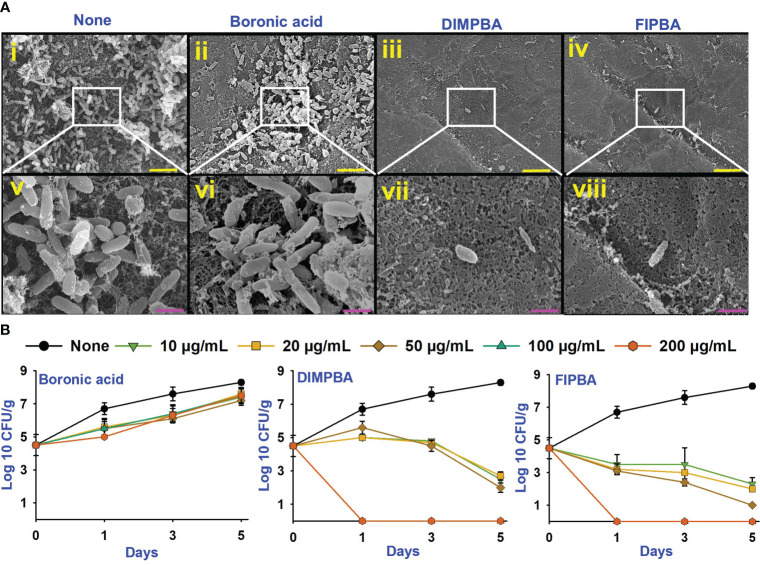
SEM images showing that the halogenated acids eradicate bacteria on squid surfaces. None indicates untreated *V. parahaemolyticus* (i, v), and compounds are being treated with a concentration of 100 µg/mL of boronic acid (ii, vi), DIMPBA (iii, vii) and FIPBA (iv, viii) **(A)**. The scale bars showed in yellow and pink color correspond to measurements of 6 µm and 1.5 µm, respectively. The halogenated acids exhibited antimicrobial effectiveness in a model involving with cooked shrimp **(B)**.

*V. parahaemolyticus* can be predominately allied with shell fish and seafood, potentially threatening human health (Bonnin-Jusserand et al., 2019). Therefore, the application of DIMPBA and FIPBA at 100 μg/mL, can impede the biofilm development by *V. parahaemolyticus* on squid surfaces. Based on the observed results, DIMPBA and FIPBA reduced the bacterial clusters detected on the squid surfaces. On the other hand, the untreated or boronic acid-treated samples exhibited the formation of cell clusters, accompanied by the presence of slimy substances produced by *V. parahaemolyticus* (**Figure 4A**).

In addition, this study assessed the antimicrobial and preservation efficacy of halogenated acids using a cooked shrimp for 5 days. Boronic acid at 10–200 μg/mL did not affect the colony-forming units (CFUs) of *V. parahaemolyticus*. Nevertheless, the DIMPBA and FIPBA compounds exhibited bactericidal activity when administered at 100 and 200 μg/mL, respectively (**Figure 4B**). In the experiment, the occurrence of *V. parahaemolyticus* in the shrimp was not visible after one day of treatment with DIMPBA and FIPBA at 100 and 200 μg/mL, respectively.

### 3.5 ADME profiling of DIMPBA and FIPBA

The in silico ADME profiling of both DIMPBA and FIPBA was carried out. The Lipinski’s Rule of Five (Boya et al., 2022) was followed by the DIMPBA and FIPBA solutions (**Supplementary Table 1**). They showed acceptable permeability of the skin, as well as permeability of the brain barrier and adsorption in the human digestive tract. Similarly, there is no indication of acute toxicity to fish or carcinogenicity in mice detected in the ADME profile. These drugs were suitable for use with the rat models being tested. **Supplementary Table l** contains a listing of all the extensive ADME parameters that were investigated.

## 4 Discussion

Biofilm development by *V. parahaemolyticus* on seafood surfaces or in food processing environments is an adaptation technique that bacteria utilize to survive in harsh environments (Yildiz and Visick, 2009). *Vibrio* species are prevalent in aquatic habitats. Some free-living *Vibrio* species may form harmful or symbiotic partnerships with eukaryotic hosts.

The boronic acid is the vital micronutrient for plants and shows biological effects binding site to the *cis-*OH groups that has been present in the cell membrane (Bolaños et al., 2004). Although boron exhibits mild bactericidal effects against numerous fungi and bacteria, there remains uncertainty regarding its efficacy against certain microorganisms (Hernandez et al., 2013). In recent years, the phenylboronic acid moiety has considerable interest as a potential antibacterial agent (Zheng et al., 2021) that may inhibit biofilm development by organisms, such as *V. parahaemolyticus* and other bacterial species. Phenylboronic acid has the potential to serve as a protecting group for diols and diamines. Additionally, it can be utilized in the regioselectively halodeboronation process, employing aqueous iodine, bromine, or chlorine (Martinko et al., 2022) used as antimicrobial agents in biology schemes (Wang et al., 2022) and antibacterial activity (Trippier and McGuigan, 2010; Cicek et al., 2019).

In the present study, various halogenated acids have distinct antimicrobial efficacy against *V. harveyi* and *V. parahaemolyticus* (**Table 1**). Two active boronic acids, i.e., 3,5-diiodo-2-methoxyphenylboronic acid (DIMPBA) and 2-fluoro-5-iodophenylboronic acid (FIPBA), exhibited antimicrobial activity and bactericidal activity at high concentrations. On the other hand, it was observed that the boronic acid did not exhibit inhibitory effects on their growth and biofilm formation (**Figures 1A, B**). Moreover, the 31 halogenated acid derivatives exhibited an MIC greater than 500 μg/mL, while its derivatives DIMPBA and FIPBA demonstrated a significantly lower MIC of 100 μg/mL (**Table 1**). Previously, the antibacterial activity of halogenated indole against *A. baumannii* was influenced by the presence of iodine atoms at the C5 position (Raorane et al., 2020). Furthermore, 5-chloro-2-methyl indole, 4-chloroindole, and 5-chloroindole had inhibitory effects on various virulence factors of *V. parahaemolyticus* and *uropathogenic E. coli* (Sathiyamoorthi et al., 2021; Boya et al., 2022). The MICs of DIMPBA and FIPBA demonstrated superior antimicrobial activity compared to quercetin at concentrations of 220 μg/mL against *V. parahaemolyticus* (Roy et al., 2022). Furthermore, it demonstrated superior potency compared to pentacyclic triterpenoids and exhibited MIC values ranging from 100–1000 μg/mL against *V. cholerae* (Bhattacharya et al., 2020). The present results revealed that the antimicrobial properties of DIMPBA and FIPBA mainly contributed to the biofilm suppression. This effect was dose-dependent when these compounds were tested against *V. parahaemolyticus* (**Figure 1C**). Moreover, the growth curve showed that two hits exhibited bactericidal activity at 100 and 200 μg/mL (**Figures 1D**, **4B**).

Compared to the untreated and boronic acid groups, the active DIMPBA and FIPBA at 10-200 μg/mL reduced thickness of the biofilm (**Figure 2A**). Motility plays a crucial virulence factor that plays a significant role during the adhesion and biofilm formation of various pathogenic bacteria (Liu et al., 2023). It facilitates the movement of bacterial cells towards nutrient-rich environments or aids in avoiding environmental stresses (Shi et al., 2023). Swimming motility relies on the flagellum (De La Fuente-Núñez et al., 2012). Therefore, DIMPBA and FIPBA effectively suppressed biofilm formation by impeding the motility of *V. parahaemolyticus* (**Figures 2B, C**). Moreover, SEM confirmed the reduction in biofilm formation, as evidenced by the observed reduction of DIMPBA and FIPBA in the aggregation of the planktonic cells of *V. parahaemolyticus* were observed (**Figure 2D**).

Fimbria attachment is crucial to bacterial adherence and the migration in the gut, as well as to the pathogenicity of many bacteria (Connell et al., 1996; Karam et al., 2018). The adaptive virulence approach in bacteria like *V. parahaemolyticus* and additional *Vibrio* species has been associated to fimbrial activity (Muthukrishnan et al., 2019; Karan et al., 2021). Therefore, the two active DIMPBA and FIPBA exhibit diminished fimbrial activity from the bacterial surfaces of *V. parahaemolyticus* (**Figure 3A**). The hydrophobicity of bacteria may vary according to strain and by environmental factors, such as temperature, nutrition availability, growth phase, and growth stage. The findings showed that *V. parahaemolyticus* adhered strongly to hydrocarbons (**Figure 3B**). Moreover, the charges on the bacterial cell fluctuates under functional circumstances, affecting how bacteria adhere to surfaces. Most *V. parahaemolyticus* cells are negatively charged and attracted to positively charged bacterial surfaces (Mizan et al., 2016). In addition, *V. parahaemolyticus* biofilm growth is also linked to the proteins and flagellar on the outer layer membrane and to the hydrophobicity of the cell surface (Mizan et al., 2016; Su et al., 2023). Particularly, the increase in adherence can be attributed to the electron-withdrawing the nature of halogen groups and the subsequent enhancements in membrane permeability and hydrophobicity when the DIMPBA and FIPBA concentration was increased from 10 to 200 μg/mL (**Figure 3B**). The halogen distribution of the parent moiety may also alter the hydrophobicity of halogenated drugs. The halogenated peptide with two bromine atoms on the phenyl rings instead of all rings was hydrophobic sufficient to kill resistant *P. aeruginosa* (Molchanova et al., 2020). Extracellular proteases help the infection move into host cell tissues, break down the amino acids for growth and survival, and digest harmful proteins (Shinoda, 2011). The classification of proteases is determined by their functional group, which includes cysteine proteases, serine proteases, metalloproteases, and aspartate proteases (Duarte et al., 2016). Hence, these findings suggest that the compounds DIMPBA and FIPBA effectively inhibit protease production and mitigate the pathogenesis associated with *Vibrio* infection (**Figure 3C**). The observed effects encompassed the suppression of biofilm formation, restriction of motility, inhibition of curli and fimbria production, reduction in protease activity, and modulation of cell surface hydrophobicity (Sathiyamoorthi et al., 2021; Boya et al., 2022).

Tryptophan breakdown produces indole, a signaling molecule in many bacteria (Defoirdt, 2023). This investigation confirms that indole diminishes *E. coli* and *V. parahaemolyticus* pathogenicity (Lee et al., 2007; Sathiyamoorthi et al., 2021). Many bacteria use the tryptophan breakdown product indole as a signaling chemical (Shin et al., 2023). The enzyme tryptophanase (TnaA), which synthesizes indole from tryptophan, also requires Na^+^ ions. An increase in Na^+^ ions may explain the indole-negative exhibit the phenotype and showed lower virulence in a *V. cholerae* oxaloacetate decarboxylase mutant (Coppens et al., 2023). Furthermore, pH plays a substantial role in indole production (Mueller et al., 2009). The production of indole in *E.coli* is suppressed by a low pH environment (Han et al., 2011). At pH 9, TnaA was observed to be one of the most highly induced proteins, as reported previously (Lee et al., 2007; Çam and Brinkmeyer, 2020). Therefore, DIMPBA and 2F5IPBA boosted indole synthesis when the pH was increased from 5 to 9. In contrast, indole synthesis decreased when the halogenated derivative concentration was increase from 10 to 200 μg/mL (**Figure 3D**). The dispersion experiment showed that halogenated acids and their derivatives dose-dependently removed preformed biofilm. For example, halogenated acids at 50 μg/mL did not removed biofilms while at 100 and 200 μg/mL, the active chemicals DIMPBA and FIPBA dispersed matured biofilm formation (**Figure 3E**).

*V. parahaemolyticus* can inhibit biofilm production on seafood surfaces, notably squid and shrimp (Bonnin-Jusserand et al., 2019). The use of 4-fluoroindole, 6-bromoindole, 7-bromindole, 7-iodoindole, and 5-iodoindole analogs improved the survival of brine shrimp that could tolerate *V. campbellii* infection (Zhang et al., 2022). This present investigation showed that DIMPBA and FIPBA prevent pathogenic bacteria from biofilm formation in the squid and shrimp models (**Figures 4A, B**). The ADME characteristics of the drug-like ligands will be assessed utilizing Lipinski’s criteria, and they had no adverse effects in acute fish and mouse models. The pharmacokinetic properties of a potential drug candidate need to be assessed early in the research and development process to minimize the risk of late-stage attrition.

## 5 Conclusions

This paper reported the effectiveness of two active boronic acid derivatives as antibacterial, antibiofilm, and maybe anti-pathogenic agents against *V. parahaemolyticus* on the cell surfaces that originate into interface with seafood. DIMPBA and FIPBA considerably reduced the viable bacterial cells, disrupted cell-to-cell contacts and preformed biofilms, and lowered the expression of virulence linked to motility, pathogenicity, and indole production. The hit halogenated derivatives might be produced as an alternative technique to control the biofilm formation of *V. parahaemolyticus* in food-contact of bacterial surfaces, reducing the risk factor of foodborne sickness triggered by this pathogen.

## Data availability statement

The raw data supporting the conclusions of this article will be made available by the authors, without undue reservation.

## Author contributions

ES: Investigation, Methodology, Writing – original draft. JL: Conceptualization, Funding acquisition, Project administration, Resources, Writing – review & editing. JL: Conceptualization, Funding acquisition, Project administration, Resources, Writing – review & editing.
