# Supplementary material for: Antibacterial and antibiofilm activity of halogenated phenylboronic acids against Vibrio parahaemolyticus and Vibrio harveyi
Source: Front Cell Infect Microbiol. 2024 Mar 28;14:1340910. doi: 10.3389/fcimb.2024.1340910 (PMC11007048; doi:10.3389/fcimb.2024.1340910)
Supplement: Supplementary file 1 [file DataSheet_1.docx]

**Supplementary Material**

**Antibacterial and antibiofilm activity of halogenated phenylboronic acids against *Vibrio* *parahaemolyticus* and *Vibrio harveyi***

Ezhaveni Sathiyamoorthi, Jin-Hyung Lee, and Jintae Lee*

^a^School of Chemical Engineering, Yeungnam University, 280 Daehak-Ro, Gyeongsan, 38541,

Republic of Korea

*Corresponding Author

E-mail: jtlee@ynu.ac.kr. Tel.: +82-53-810-2533. Fax: +82-53-810-4631.

**Supplementary Table 1:** ADME profiles of the two halogenated acids selected in this study. It showed the combined ADME properties of the two halogenated acids from the online web servers, including PreADMET, Molinspiration, and GUSAR, accessed on 16 August 2023.

| **Property** | **DIMPBA** | **FIPBA** |
| --- | --- | --- |
| **Lipinski's rule of five** | Suitable | Suitable |
| **Lipinski’s rule of five violations** | 0 | 0 |
| **Plasma protein binding** | 91.883650 | 14.047591 |
| **blood-brain barrier permeability** | 2.10884 | 1.91903 |
| **Skin permeability** | -3.78339 | −2.99125 |
| **Human intestinal absorption(HIA)** | 98.318031 | 98.057505 |
| **Caco 2** | 22.5982 | 20.8571 |
| **Mouse carcinogenicity** | Out of range | Out of range |
| **Acute fish toxicity (medaka)** | 0.00178556 | 0.0228331 |
| **Acute fish toxicity (minnow)** | 0.00326098 | 0.0155183 |
| ***In-Vitro* hERG inhibition** | Medium risk | Medium risk |
| **miLogP** | 3.06 | 2.16 |
| **mol volume** | 211.65 | 167.04 |
| **TPSA** | 49.69 | 40.46 |
| **GPCR ligand** | −0.84 | −0.88 |
| **Ion channel modulator** | −0.34 | −0.31 |
| **Kinase inhibitor** | −0.44 | −0.52 |
| **Nuclear receptor ligand** | 0.09 | 0.10 |
| **Protease inhibitor** | −0.26 | −0.21 |
| **Enzyme inhibitor** | 0.74 | 0.77 |
| **Rat IP LD50 classification** | Class 4 in AD | Class 4 in AD |
| **Rat IV LD50 classification** | Class 4 in AD | Class 4 in AD |
| **Rat oral LD50 classification** | Class 4 in AD | Class 4 in AD |

**
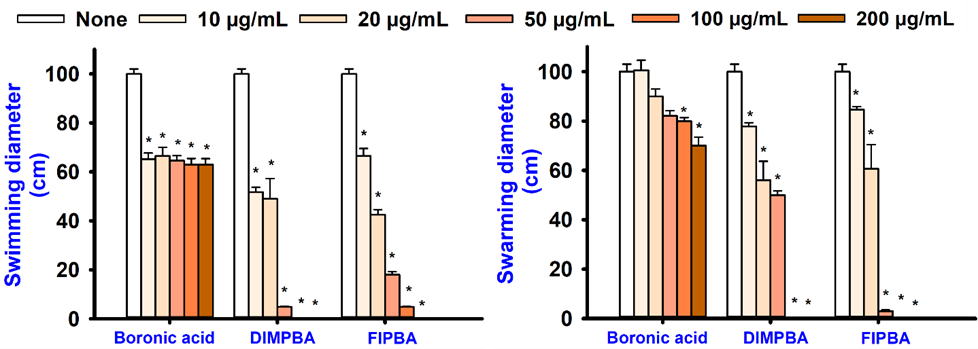
Supplementary Figure 1**: Diameter for swimming and swarming motility for boronic acid and selected hit compounds DIMPBA and FIPBA


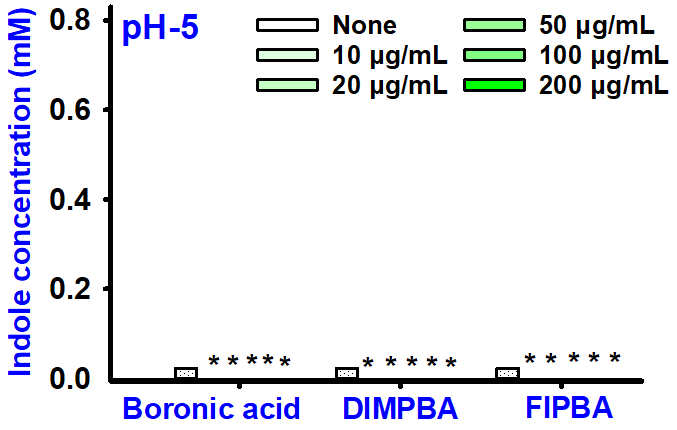


**Supplementary Figure 2**: Indole production at pH 5. The asterisk (*) denotes statistical significance at a significance level of p < 0.05.
